# Upcycling eggshell waste into sustainable and portable chiral membranes for the physical separation and electrocatalytic deracemization of enantiomers

**DOI:** 10.1039/d6ra03698h

**Published:** 2026-07-03

**Authors:** Malinee Niamlaem, Sara Grecchi, Mariangela Longhi, Serena Arnaboldi

**Affiliations:** a Università degli Studi di Milano, Department of Chemistry Via Golgi 19 Milan 20133 Italy serena.arnaboldi@unimi.it

## Abstract

The preparative separation and synthetic deracemization of enantiomers are conventionally dominated by resource-intensive chromatographic methods and chemically wasteful asymmetric syntheses, posing significant challenges to Green Chemistry principles. Herein, we present a highly sustainable, electrically switchable chiral bio-hybrid device, achieved by upcycling globally abundant avian eggshell biomineral waste (CaCO_3_). Exploiting the raw, heterogeneously sized microparticles as a binder-free, hierarchically porous structural scaffold, we engineered a “structural multiplier” that drastically amplifies the stereospecific interactions of an electrodeposited inherently chiral oligomer. Integrated into a miniaturized, solvent-efficient continuous-flow setup operating with microliter volumes and green extraction media (heptane), the bio-hybrid interface completely transcends conventional physical filtration. We demonstrate that by simply tuning the externally applied DC bias, the membrane's operational logic toward carvone enantiomers is dynamically inverted. At a moderate anodic bias (0.7 V), it acts as a passive thermodynamic affinity filter, while at 1.3 V, it rigidifies into an absolute steric gatekeeper. Most remarkably, poised at an intermediate hyper-reactive regime (1.1 V), the system operates as a solid-state electrocatalytic reactor capable of actively breaking racemic equilibriums. Through an iterative, multi-pass continuous-flow process, the membrane actively interconverted the unfavoured enantiomer into the favoured antipode, upgrading a strict 50 : 50 racemic mixture to an exceptional >99% enantiomeric excess (ee) using solely localized electrical perturbation. This work seamlessly merges zero-cost biomineral valorization with advanced molecular electrochemistry, offering a low-energy, reagent-free paradigm that bridges green enantioseparation and sustainable asymmetric synthesis.

## Abstract 2

Eggshell waste is upcycled into a robust bio-hybrid chiral membrane. Tuning the electrical bias switches its logic from a passive filter to an active reactor, driving racemates to >99% ee in continuous flow.

## Graphical abstract

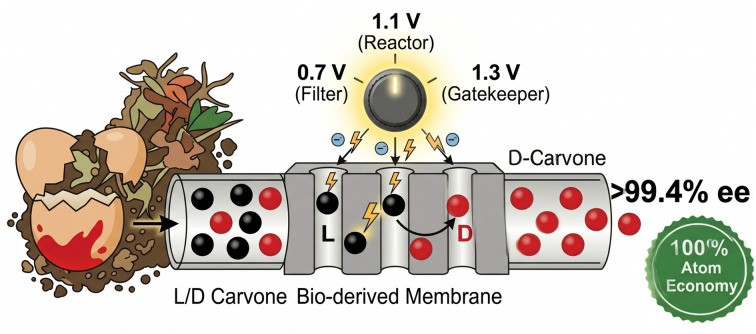

## Introduction

The analytical discrimination and physical separation of enantiomers represent a cornerstone of modern chemical science, with profound implications spanning from the safety of pharmaceutical formulations to the organoleptic quality of food products.^1,2^ Chirality is a fundamental pillar of biological recognition; consequently, the two enantiomers of a chiral molecule often exhibit vastly different pharmacological profiles, toxicological effects, or sensory perceptions.^3–5^ A quintessential example is found in the monoterpene carvone: its (*S*)-(+)-enantiomer is the primary constituent of caraway oil, while the (*R*)-(−)-antipode defines the characteristic aroma of spearmint,^5^ highlighting how chirality alone can dictate macroscopic function. The ability to separate such species with high efficiency is not merely a technical requirement but a necessity for ensuring the integrity of high-value chemical products.^6–8^ Over the past decades, chiral chromatography, particularly high-performance liquid chromatography (HPLC) with chiral stationary phases (CSPs), has become the dominant analytical and preparative approach for enantioseparation.^8–12^ Despite its effectiveness, this paradigm is increasingly questioned when evaluated through the lens of Green Analytical Chemistry (GAC). Conventional chromatographic methods are intrinsically resource-intensive, requiring large volumes of organic solvents, energy-demanding instrumentation, and stationary phases often derived from petroleum-based polymers or complex synthetic routes. These drawbacks are amplified when chiral analysis must be performed outside centralized laboratories, for instance, in quality control along supply chains, on-site screening, or decentralized pharmaceutical manufacturing. In response to these limitations, the analytical community is actively exploring alternative strategies that are more compatible with sustainability, miniaturization, and continuous-flow processing. Among these, membrane-based enantioseparation has emerged as a particularly attractive concept. Unlike batch or column-based techniques, chiral membranes enable continuous transport and discrimination processes, drastically reduce solvent consumption, and can be readily integrated into compact microfluidic devices.^13–15^ Historically, chiral membranes have utilized a variety of scaffolds, including cellulose derivatives, molecularly imprinted membranes (MIMs),^16–19^ Metal–Organic Frameworks (MOFs),^20–22^ and Covalent Organic Frameworks (COFs).^23,24^ However, despite impressive performances, many of these systems still face practical barriers. Complex synthesis protocols limited mechanical robustness, sensitivity to solvent-induced swelling, and reliance on expensive or non-renewable building blocks remain significant challenges. There is a clear and unmet need for chiral functional scaffolds that combine high stereoselectivity, structural robustness, scalability, and authentic sustainability. In this context, the upcycling of bio-waste materials offers a compelling and timely opportunity. Avian eggshells represent one of the most abundant and underutilized byproducts of the food industry, with millions of tons generated annually worldwide. While the inner proteinaceous membrane has been widely studied, the macroscopic calcified shell itself, composed overwhelmingly of highly crystalline, biomineralized calcium carbonate (CaCO_3_), is a remarkably sophisticated and often overlooked natural scaffold.^25,26^ Following the deliberate removal of the inner membrane, the ground calcified shell microparticles exhibit intrinsic hierarchical micro-porosity, outstanding mechanical rigidity, and high chemical stability. From an analytical perspective, this pulverized biomineral matrix offers decisive advantages over conventional two-dimensional substrates or soft synthetic polymer supports. The heterogeneously sized calcium carbonate microparticles naturally pack to create a highly tortuous, three-dimensional labyrinthine micro-environment. This complex geometry acts as a “structural multiplier”: it significantly increases the specific surface area and disrupts rapid laminar diffusion, enforcing an extended contact time between permeating analytes and the functionalized surface. Furthermore, its inherent stability allows for the direct anchoring of electroactive materials without the need for cytotoxic adhesion promoters or synthetic polymeric binders. Importantly, utilizing this raw, unmodified biomineral directly addresses circular economy goals by transforming an otherwise discarded bio-waste into a high-added-value structural scaffold. While the biomineral provides an ideal sustainable backbone, enantioselective performance critically depends on the nature of the chiral selector. In this regard, inherently chiral materials represent a conceptual breakthrough. As demonstrated in recent studies, inherently chiral oligomers and polymers derive their chirality not from localized stereogenic centers, but from the overall torsion and topology of their π-conjugated backbone.^27,28^ This design principle leads to an exceptionally high density of chiral recognition sites and to strong, energy-differentiated interactions with chiral guests. Among these systems, the atropisomeric oligothiophene BT_2_T_4_ (2,2′-bis(2,2′-bithiophene-5-yl)-3,3′-bibenzothiophene) stands out as a robust and versatile inherently chiral selector.^29,30^ Previous work has demonstrated its outstanding enantiodiscrimination capabilities in unconventional electrochemical applications, such as wireless separation, unplugged asymmetric synthesis and self-standing electroactive membranes.^31–34^ Building on these advances, the present work introduces a novel class of sustainable, electrically switchable chiral devices obtained by electrochemically functionalizing the CaCO_3_ biomineral network of eggshell waste with the inherently chiral BT_2_T_4_ oligomer.

By coupling a highly tortuous, waste-derived inorganic scaffold with a state-of-the-art electroactive chiral selector, we bridge two traditionally distant domains: circular bio-waste upcycling and high-level stereochemical modulation. Herein, we report the binder-free fabrication, characterization, and application of oligo-BT_2_T_4_@eggshell bio-hybrids in a miniaturized continuous-flow setup for the management of carvone enantiomers.

Crucially, we demonstrate that the combination of the biomineral's tortuous porosity and the polymer's electroactive backbone allows the membrane to completely transcend basic physical separation. By dynamically tuning the externally applied electrical potential, this smart bio-hybrid can be seamlessly switched between acting as a passive thermodynamic affinity filter, an absolute steric gatekeeper, or, most remarkably, an active solid-state electrocatalytic reactor capable of continuous-flow deracemization.

In the broader perspective of Green Chemistry, this approach exemplifies how advanced molecular design and zero-cost biomineral upcycling can converge to deliver low-energy, highly sustainable alternatives to traditional preparative chiral chromatography and complex asymmetric synthesis.

## Experimental

### Chemicals and reagents

All the reagents, chemicals, and solvents used in this work were of analytical grade. Lithium perchlorate (LiClO_4_, 99%), D-(+)- and L-(−)-carvone, doxorubicin, water (HPLC grade), and heptane (HPLC grade) were purchased from Sigma Aldrich. Anhydrous acetonitrile (ACN, 99%, HPLC grade) was obtained from Honeywell Riedel-de-Haën™. All chemicals were used as received without further purification. The enantiopure inherently chiral oligomer, (*S*)- or (*R*)-2,2′-bis[2-(5,2′-bithienyl)]3,3′-bithianaphthene ((*S*)-BT_2_T_4_ or (*R*)-BT_2_T_4_, Fig. S1), as well as the racemic monomer, were synthesized following previously published methodologies.^29,30^

### Characterizations

Scanning electron microscopy (SEM) experiments were carried out using a FEI QUANTA 450 microscope at 20.0 kV to assess the heterogeneous microparticle morphology of the biomineral scaffold and the subsequent conformal coating of the inherently chiral oligomer. The morphological investigation was performed on both the raw pulverized eggshell waste (Fig. S2a) and the final oligo-BT_2_T_4_@eggshell bio-hybrid membrane (Fig. S2c). Fourier transform infrared (FTIR) spectroscopy was performed with a Jasco spectrometer (FT/IR-4600) in a wavenumber range of 500–4000 cm^−1^ (200 scans) to verify the chemical composition of the eggshell waste and directly compare it with commercial CaCO_3_ (Fig. S2b), as well as to confirm the successful integration of the polymer after modifications. The oligo-BT_2_T_4_@eggshell membrane, after detachment from the electrode, was visually monitored using a CCD camera (CANON EOS R7, Objective Canon Macro Lens 100 mm 1 : 2.8).

Specific surface areas were obtained from N_2_ adsorption/desorption isotherms at 77 K using a Micromeritics Tristar II 3020 apparatus together with instrumental software (Version 1.03) and applying Brunauer–Emmett–Teller (BET). Sample powders were heat-treated (*T* = 60 °C, 24 h, N_2_) before the analysis to remove adsorbed water.

Spectroelectrochemical measurements were performed to investigate the potential-induced electronic transitions of the bio-hybrid composite under operational conditions. The optical absorption spectra were recorded in the near-ultraviolet, visible, and near-infrared regions (from 300 to 1400 nm) using a Shimadzu UV-Vis-NIR spectrometer coupled with a PalmSens potentiostat. The experiments were conducted at room temperature in a custom-built, three-electrode, one-compartment quartz cell. An indium tin oxide (ITO) substrate, modified with either the pristine eggshell powder or the electrodeposited oligo-BT_2_T_4_@eggshell network, was employed as the working electrode. A platinum (Pt) sheet and a silver (Ag) wire pseudo-reference were used as the counter and reference electrodes, respectively. The cell was filled with a 0.1 M lithium perchlorate (LiClO_4_) solution in acetonitrile (ACN) to serve as the supporting electrolyte. To evaluate the different operational modes, a constant DC bias (0.0 V, 0.7 V, 1.1 V, and 1.3 V) was sequentially applied to the working electrode. Prior to each spectral acquisition, the applied potential was held constant for a sufficient equilibration period (∼1 minute) to allow the system to reach an electrochemical steady state and ensure a stable and uniform doping level across the polymer bulk.

### Electrosynthesis and electrochemical characterization of inherently chiral BT_2_T_4_ on eggshell biomineral scaffold

**Fig. 1:**
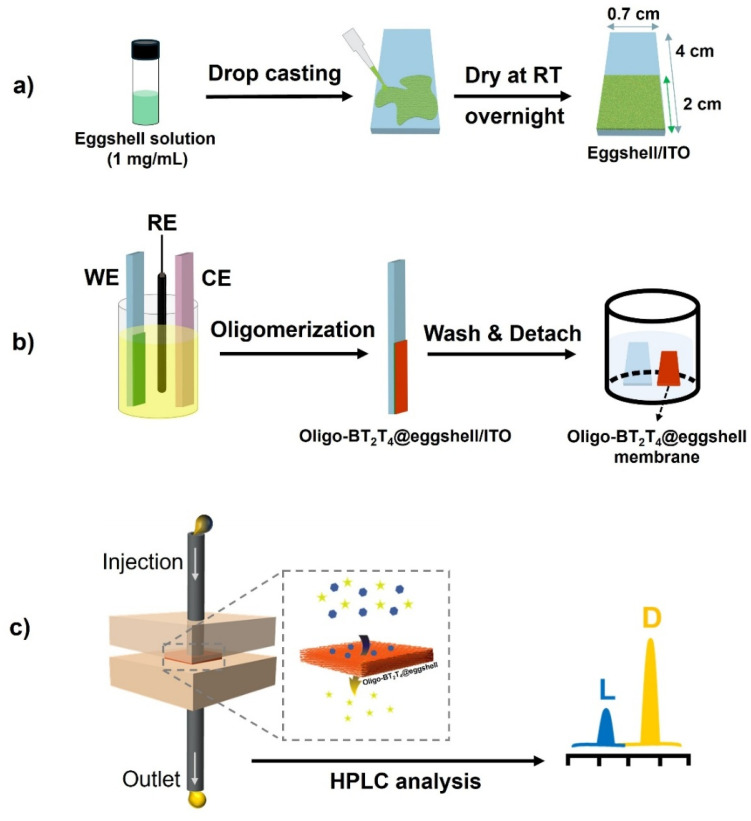
Schematic overview detailing the binder-free fabrication process and the miniaturized continuous-flow analytical setup for the sustainable oligo-BT_2_T_4_@eggshell bio-hybrid device. (a) Preparation of the structural biomineral scaffold: an aqueous suspension of upcycled, heterogeneously sized eggshell microparticles (1 mg mL^−1^) is drop-cast onto an indium tin oxide (ITO) substrate and dried at room temperature overnight, generating a stable, highly tortuous, and completely binder-free inorganic layer. (b) Electrochemical functionalization and membrane recovery: anodic potentiodynamic electrosynthesis of the inherently chiral BT_2_T_4_ network utilizing a standard three-electrode cell configuration. A subsequent brief immersion in deionized water allows the smooth detachment of the physically encapsulated composite film, yielding a robust, free-standing bio-hybrid chiral membrane. (c) Integration into the miniaturized continuous-flow setup: the free-standing membrane is securely sandwiched between two customized silicon rings and interfaced with microcapillaries. The chiral feed (*e.g.*, carvone enantiomers) injected at the inlet is dynamically forced through the electroactive biomineral labyrinth, and the processed permeate is continuously collected at the outlet for subsequent quantitative stereochemical evaluation *via* high-performance liquid chromatography (HPLC).

The bio-hybrid membranes of oligo- BT_2_T_4_ and eggshell waste were prepared following a binder-free, two-step procedure as shown in Fig. 1a and b. First, waste eggshells were collected from household solid waste in Milan, Italy. The collected shells were thoroughly washed with deionized (DI) water to remove surface impurities. Crucially, the inner proteinaceous membrane was carefully removed manually and strictly discarded. The remaining calcified macroscopic shells were repeatedly rinsed with DI water and subsequently dried in an oven at 100 °C overnight. After drying, the inorganic shells were finely ground using a mortar to obtain a highly heterogeneous biomineral eggshell powder. The biomineral suspension was prepared by dispersing 1 mg of the obtained powder in 1 mL of DI water, followed by sonication for 10 min to achieve a homogeneous dispersion. To prepare the electrode, 120 µL of the eggshell suspension was drop-cast onto a 0.7 × 4 cm ITO substrate, covering a 2 cm length. The electrode was then dried under ambient conditions overnight to form a highly stable, tortuous support without using any synthetic chemicals or binders, denoted as eggshell/ITO (Fig. 1a). Second, the electrosynthesis of the enantiopure oligo-(*S*)- and oligo-(*R*)- BT_2_T_4_ (as well as the racemic control film) was achieved by cyclic voltammetry in a conventional three-electrode cell composed of the eggshell/ITO, a Pt sheet, and an Ag/AgCl, as working, counter, and reference electrodes, respectively. The potentiodynamic oligomerization was performed by scanning from 0.0 V to 1.3 V *vs.* Ag/AgCl at 0.05 V s^−1^ for 108 cycles, in a 0.1 M LiClO_4_ and 0.75 mM monomer ACN solution. After oligomerization, the resulting membrane was detached from the ITO surface by brief immersion in DI water to yield a free-standing chiral film ready for filtration assembly, denoted as oligo-(*R*)-BT_2_T_4_@eggshell or oligo-(*S*)-BT_2_T_4_@eggshell (Fig. 1b). Electrochemical impedance spectroscopy (EIS) was further used to describe the interface properties of the hybrid oligo-BT_2_T_4_@eggshell/ITO, bare eggshell/ITO, and bare oligo-BT_2_T_4_/ITO electrodes. The measurements were carried out in an aqueous solution containing 0.1 M LiClO_4_. The data were recorded in the frequency range from 0.1 to 100 000 Hz with a perturbation amplitude of 10 mV. The obtained impedance (*Z*) data were expressed as Nyquist plots (negative imaginary impedance-*Z*″ *versus* the real part of the impedance *Z*′).

### Enantiorecognition tests

Potentiodynamic measurements of two chiral probes, doxorubicin (1 mM in pH 4 buffer) and d- or l-carvone (4 mM in 0.1 M LiClO_4_ aqueous solution +100 µL ACN), were performed on the obtained oligo-(*R*)-BT_2_T_4_@eggshell or oligo-(*S*)-BT_2_T_4_@eggshell electrodes. The electrochemical characterization was conducted by cyclic voltammetry using an AUTOLAB potentiostat-galvanostat PGSTAT101 (Metrohm Lab Instruments) coupled with a three-electrode cell utilizing the modified working electrode, a Pt sheet counter, and an Ag/AgCl reference electrode. The voltammograms were recorded in a potential range from 0 to 1.5 V *vs.* Ag/AgCl at a scan rate of 5 mV s^−1^ for doxorubicin. To specifically evaluate the impact of the biomineral's morphological packing on enantiorecognition, four distinct particle size fractions of the eggshell powder (heterogeneous un-sieved, >100 µm, 50–100 µm, and <50 µm) were prepared and tested using doxorubicin (Fig. S3). These specific size-controlled fractions were successfully isolated by utilizing a series of calibrated molecular sieves to ensure a precise and reproducible classification of the pulverized biomineral matrix. For l- or d-carvone, the tests were recorded from 0 to 1.6 V *vs.* Ag/AgCl at 50 mV s^−1^. Derivative cyclic voltammograms were also calculated to better highlight the baseline thermodynamic peak potential separations for the carvone enantiomers (Fig. S4). For all tests, the active BT_2_T_4_ films were prepared using 5 electropolymerization cycles for doxorubicin and 108 cycles for the carvone enantiorecognition.

### Chiral separation and high-performance liquid chromatography (HPLC) analysis

For the chiral membrane separation, the miniaturized continuous-flow cell was assembled by securely sandwiching the oligo-BT_2_T_4_@eggshell membrane between two customized silicon rings (internal diameter 1.5 mm, thickness 1 mm) (Fig. 1c). Two capillaries (1.5 mm internal diameter) were interfaced at the inlet and outlet to allow for fluid transport. The total internal volume (dead volume) of the assembled cell was estimated to be approximately 25 µL. Instead of utilizing external pumping systems, the 40 µL chiral probe was introduced *via* manual injection at the inlet capillary, and the permeate was allowed to pass through the biomineral membrane *via* gravity-assisted flow.

Then, 40 µL of the chiral probe (l-carvone, d-carvone, or a strictly 50 : 50 racemic carvone) was injected into the capillary. All the permeating fractions were continuously collected from the outlet side of the capillary using a micropipette (5 µL of each fraction). The products were subsequently extracted using 1.5 mL of heptane as a green extraction solvent.

For the reusability tests, the bio-hybrid membrane was regenerated between consecutive filtration cycles by flushing the setup with 1.5 mL of heptane. This procedure ensures the complete removal of any enantiomer previously retained within the biomineral matrix, resetting the chiral recognition sites. Following the washing step, the specific 1.3 V voltage was re-applied for 5 minutes prior to the next injection to ensure a stable p-doping state and reproducible operational conditions. For the chiral HPLC analyses, all measurements were carried out with an HPLC equipment (Agilent 1260 Infinity II) coupled with a Daicel CHIRALPAK IG-3 column in isocratic reverse phase conditions. The HPLC analyses of d-, l-, or racemic carvone were performed by injecting 5 µL of each heptane extract into the chiral column with ACN/H_2_O (50 : 50) as eluent at a flow rate of 1 mL min^−1^. The photodiode array (PDA) detector was operated at a wavelength of 236 nm, and the column temperature was strictly set at 20 °C. In all experiments, the racemic mixture and each individual enantiomer were analyzed separately and simultaneously under identical conditions to enable an accurate comparison of the enantioseparation results and ensure the correct assignment of the chromatographic peaks.

To evaluate the specific structural origin of the potential-tunable gating, analogous control experiments were conducted using the counterpart oligo-(*S*)-BT_2_T_4_@eggshell membrane as well as a non-enantioselective oligo-Racemic-BT_2_T_4_@eggshell control membrane across different applied potentials.

The iterative deracemization of the racemic mixture *via* the multi-pass continuous-flow approach was accurately tracked by evaluating the enantiomeric ratios across consecutive electrocatalytic filtration cycles. The final enantiomeric excess (ee%) of the collected fractions was mathematically calculated using the following fundamental equation:
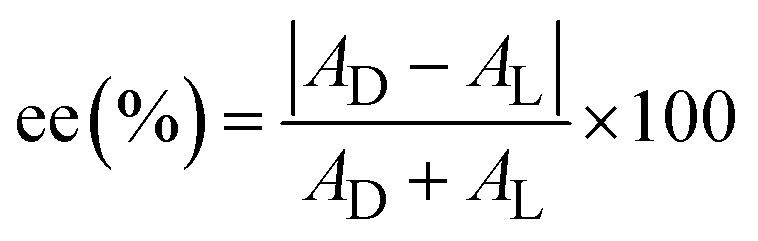
where *A*_D_ and *A*_L_ are the integrated HPLC peak areas of the d- and l-carvone enantiomers, respectively.

All experimental measurements were performed in triplicate (*n* = 3), and results are reported as the mean value ± standard deviation. Error bars representing the statistical deviation have been included in the corresponding figures.

## Results and discussion

### Fabrication and morphological/chemical characterization of the bio-hybrid interface

The development of high-performance enantioselective interfaces in analytical sensing requires the precise spatial organization of chiral recognition sites, a parameter that is critically dependent on the physical and chemical architecture of the supporting substrate.

In this study, naturally occurring calcified eggshell waste was utilized as a macroscopic, highly porous, and environmentally sustainable biomineral scaffold. The working electrode was prepared *via* a binder-free approach by simple drop-casting of an aqueous eggshell suspension (1 mg mL^−1^) onto an ITO substrate (Fig. 1a), followed by the potentiodynamic electropolymerization of the inherently chiral BT_2_T_4_ monomer in a conventional three-electrode cell (Fig. 1b).

This protocol generated a free-standing, physically robust oligo-BT_2_T_4_@eggshell membrane ready to be integrated into a miniaturized continuous-flow setup (Fig. 1c).

The highly granular and tortuous surface morphology of the free-standing bio-hybrid membrane, as revealed by SEM analysis (Fig. S2c), confirms the creation of a three-dimensional labyrinthine network. At higher magnifications, the images demonstrate the successful physical encapsulation of the biomineral microparticles by a dense, conformal layer of oligo-BT_2_T_4_, yielding the robust core–shell architecture responsible for both chiral recognition and chemical passivation.

To quantitatively validate the textural evolution of this biomineral scaffold upon polymer integration, N_2_ adsorption–desorption isotherms were recorded to evaluate the specific surface area and pore dimensions (Table S1, SI).

Interestingly, the specific surface area of the pristine eggshell biomaterial remains relatively low and nearly constant across all isolated particle size fractions (ranging from 1.87 ± 0.02 m^2^ g^−1^ for the <50 µm batch to 2.61 ± 0.05 m^2^ g^−1^ for the heterogeneous random-size packing).

This indicates that the macroscopic particle size distribution does not inherently alter the absolute microscopic surface area of the bare inorganic matrix, suggesting that the differences observed in electrochemical resolution among the size-selected fractions are primarily governed by the extended analyte residence time within the more tortuous diffusion pathways of the heterogeneous packing.

However, following the binder-free deposition of the oligo-BT_2_T_4_ network onto the random-size scaffold, the specific surface area of the resulting bio-hybrid composite undergoes a dramatic ten-fold increase, reaching 27 ± 2 m^2^ g^−1^. This remarkable amplification provides quantitative evidence of the “structural multiplier” effect.

The conformal electrodeposition of the oligomer does not simply passivate the biomineral surface with a flat layer; rather, it generates the highly nanostructured, three-dimensional electroactive architecture observed in SEM, which drastically expands the available active area for chiral recognition. Concurrently, a slight reduction in the cumulative pore volume (from 0.020 to 0.010 cm^3^ g^−1^) is morphologically consistent with the partial coating and filling of the macroscopic biomineral voids by the conjugated polymer network, definitively transforming the inert macro-pores into a highly active chiral labyrinth.

**Fig. 2:**
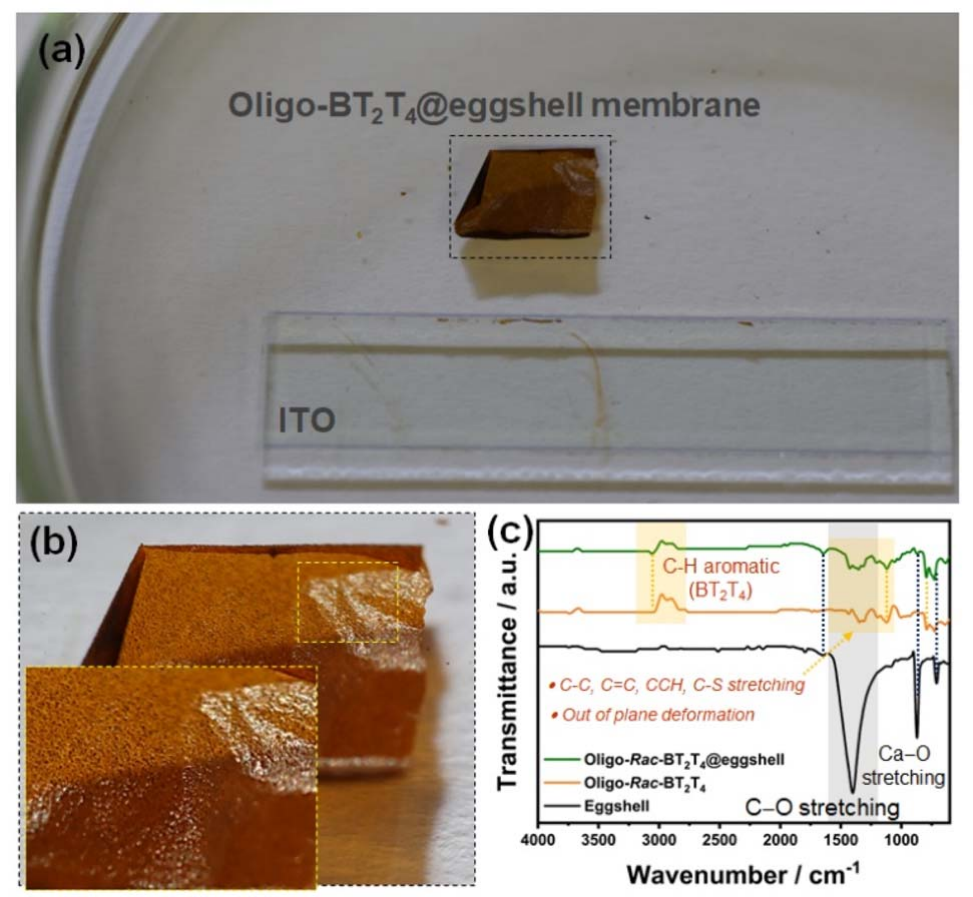
Morphological and chemical characterization of the binder-free bio-hybrid interface. (a) Low- and (b) high-magnification optical photographs demonstrating the physical integrity and the highly granular, tortuous surface morphology of the free-standing oligo-BT_2_T_4_@eggshell membrane successfully detached from the underlying ITO working electrode. The enantiopure membrane was obtained *via* cyclic voltammetry using a 0.75 mM BT_2_T_4_ monomer solution in ACN +0.1 M LiClO_4_ at 50 mV s^−1^ for 108 cycles. (c) FTIR spectra comparing the raw biogenic eggshell waste (black trace, dominated by typical CaCO_3_ stretching modes), the pure electrodeposited oligo-Racemic-BT_2_T_4_ film (orange trace), and the composite oligo-Racemic-BT_2_T_4_@eggshell membrane (green trace). The composite spectrum unequivocally confirms the successful conformal electrodeposition of the organic Π-conjugated network, evidenced by the diagnostic aromatic C–H stretching at ∼3060 cm^−1^ and skeletal C–C/C=C/C–S vibrational bands, while demonstrating the strict structural preservation of the inorganic CaCO_3_ core (dominant C–O and Ca–O stretching modes) despite the aggressive anodic oxidative conditions.

Optical characterizations of the detached membrane (Fig. 2a and b) reveal a brownish, physically independent architectural fragment. As previously demonstrated by structural investigations of mechanically ground eggshells, the un-sieved microparticles naturally pack to create a highly tortuous, three-dimensional labyrinthine network. This interconnected porosity acts as a “structural multiplier”: it avoids flat conformations and establishes a complex micro-environment that drastically increases the electrochemically effective surface area.

To definitively ascertain the chemical composition of the biogenic substrate and verify the successful integration of the inherently chiral polymer network, Fourier Transform Infrared (FTIR) spectroscopy was employed (Fig. 2c).

The pre-modification spectral signature of the pulverized eggshell waste exhibits classical biogenic mineral characteristics, aligning perfectly with pure calcium carbonate (CaCO_3_).

Three dominant vibrational modes manifest distinctly: a prominent peak at 1387 cm^−1^ corresponding to the asymmetric stretching of the carbonate C–O bond, and sharp signals at 873 cm^−1^ and 713 cm^−1^ associated with the Ca–O out-of-plane and in-plane bending vibrations, respectively. Following electropolymerization, the FTIR spectrum of the oligo-BT_2_T_4_@eggshell construct displays a complex composite fingerprint. A diagnostic absorption band emerges at 3060 cm^−1^, unequivocally assigned to the aromatic C–H stretching vibrations of the BT_2_T_4_ framework, alongside a broad multi-peak region (500–1500 cm^−1^) encompassing C–C, C=C, CCH, and C–S stretching modes.

The strict preservation of the intense CaCO_3_ peaks verifies a crucial mechanistic parameter: the aggressive anodic oxidative potential sweeps required to oligomerize the monomer do not induce structural degradation of the underlying biomineral lattice.

### Electrochemical enantiorecognition, particle size effect, and core–shell chemical passivation

The chiral recognition capability of the synthesized interfaces was initially benchmarked using cyclic voltammetry (CV) in the presence of doxorubicin enantiomers (Fig. S3). Oligo-(*R*)-BT_2_T_4_ and oligo-(*S*)-BT_2_T_4_ deposited directly onto a planar, unmodified ITO electrode served as the experimental baseline, demonstrating a moderate peak potential separation (Δ*E*_p_) of 170 mV (Fig. S3a, inset).

Strikingly, the transition to the three-dimensional, hierarchically porous oligo- BT_2_T_4_@eggshell/ITO architecture triggered a profound amplification of the chiral recognition efficiency, exhibiting a staggering peak potential separation of 300 mV. A critical sub-investigation definitively proved the role of the biomineral's physical packing. Four distinct size fractions of eggshell powder were evaluated: large (>100 µm), intermediate (50–100 µm), fine (<50 µm), and fully heterogeneous (un-sieved). Counterintuitively, the uniformly sized particles produced poor to moderate enantiomeric peak separations (70 to 100 mV). In stark contrast, the fully heterogeneous powder achieved the unparalleled 300 mV separation.

Uniformly sized particles inevitably establish highly regular interstitial voids that allow for rapid laminar diffusion. Conversely, a heterogeneous size distribution forces smaller mineral fragments to densely occupy the interstitial voids of larger granules, severely hindering mass transfer and drastically reducing the mean free path of the analyte. This tortuous micro-channel network maximizes the analyte-surface contact time, proving that strict morphological uniformity is actively detrimental to stereospecific recognition in this system. At this stage, it is crucial to highlight a remarkable secondary advantage imparted by the electrodeposited chiral network: the chemical stabilization of the biomineral support. Unprotected biogenic calcium carbonate is notoriously acid-sensitive and undergoes rapid hydrolytic dissolution with continuous CO_2_ evolution in mildly acidic media, such as the pH 4 buffer strictly required to preserve doxorubicin stability.

However, during the electrochemical tests, the bio-hybrid electrode exhibited highly stable faradaic responses with absolutely no evidence of macroscopic gas evolution or signal noise. This unexpected chemical resilience is a direct consequence of the protective nature of the chiral selector. The potentiodynamic oligomerization yields a dense, conformal polymer layer that physically encapsulates the biomineral microparticles, creating a robust “core–shell” architecture. The highly hydrophobic, π-conjugated backbone severely restricts the permeation of the aqueous acidic electrolyte, effectively acting as an impermeable passivating shield. Consequently, the oligo- BT_2_T_4_ film expands the operational pH window of the waste-derived scaffold, enabling robust chiral analysis even in hostile acidic environments.

### Mechanistic validation *via* Electrochemical Impedance Spectroscopy (EIS)

To expand the analytical scope beyond complex pharmaceuticals, the bio-hybrid system was challenged with d- and l-carvone, a classic pair of small monoterpenoid enantiomers. Baseline voltammetry demonstrated a definitive anodic peak separation of approximately 200 mV (Fig. S4).

**Fig. 3:**
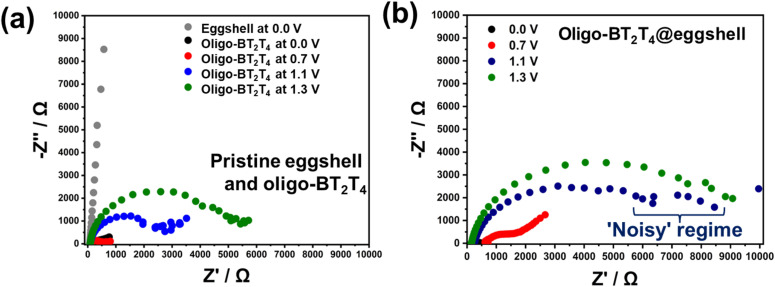
Mechanistic validation of the electrically switchable chiral interface *via* electrochemical impedance spectroscopy (EIS) and symmetry evaluation. (a) Nyquist plots establishing the comparative baseline: the pristine bare eggshell/ITO electrode at 0.0 V (grey dots, confirming the purely capacitive, structurally inert nature of the biomineral) and the planar bare oligo-(*R*)-BT_2_T_4_/ITO electrode probed under varying applied DC biases (0.0 V to 1.3 V). (b) Nyquist plots of the 3D bio-hybrid oligo-(*R*)-BT_2_T_4_@eggshell membrane under the corresponding applied potentials. The dramatic overall increase in charge transfer resistance (*R*_ct_) compared to panel (a) proves the geometrical impedance amplification provided by the tortuous biomineral matrix (“structural multiplier” effect). The distinct low-frequency scatter observed strictly at the 1.1 V bias (blue dots, highlighted as the ‘Noisy’ regime) represents the localized kinetic instability footprint arising from the active electrocatalytic interconversion of the enantiomers, physically preventing the system from reaching a steady state. All EIS measurements were performed in an aqueous 0.1 M LiClO_4_ supporting electrolyte.

To meticulously untangle the heterogeneous charge transfer mechanics and demonstrate the transition from passive sensing to active, potential-driven chiral separation, Electrochemical Impedance Spectroscopy (EIS) was performed (Fig. 3). The EIS profile of the pristine bare eggshell/ITO electrode (Fig. 3a) resolves into a nearly vertical line at low frequencies, confirming that the raw CaCO_3_ biomineral is purely capacitive and completely lacks intrinsic electroactivity or chiral-selective properties; its role is strictly structural.

When comparing the EIS profiles at the maximum applied potential (1.3 V), the pure oligo-(*R*)-BT_2_T_4_/ITO film yields a charge transfer resistance (*R*_ct_) of ∼5.5 kΩ (Fig. 3a), while the oligo-(*R*)-BT_2_T_4_@eggshell membrane exhibits an *R*_ct_ exceeding ∼10 kΩ (Fig. 3b).

This massive impedance amplification proves the “structural multiplier” effect: solvated carvone molecules are forced to navigate a geometrically complex network conformally coated with the oxidized chiral selector.

The step-wise EIS mapping of the oligo-(*R*)-BT_2_T_4_@eggshell membrane as a function of the applied DC bias (Fig. 3b) defines three distinct operational regimes:

• Passive filter mode (0.7 V): the partial *p*-doping of the thiophene backbone establishes a moderate *R*_ct_. The polymer begins to discriminate permeating molecules based strictly on structural thermodynamic affinity.

• Reactive “noisy” mode (1.1 V): the Nyquist plot exhibits noticeable low-frequency scatter (blue curve). While such behavior might initially suggest interfacial instability or bubble formation, post-operative SEM analysis (Fig. 7) rules out structural degradation, polymer delamination, or biomineral erosion. Additionally, the mild 1.1 V bias avoids macroscopic gas evolution in this specific setup. Therefore, this low-frequency perturbation can be attributed to a highly dynamic, non-steady-state electrochemical regime at the bio-hybrid interface. Rather than serving as direct proof of the chiral switch, this impedance noise represents the macroscopic electrical signature of the ongoing electrocatalytic process. It is consistent with the continuous, non-equilibrium cycles of molecular adsorption, surface-mediated structural reorganization (tautomerization), and subsequent desorption of the carvone species, which physically prevent the system from reaching the steady state required for standard EIS mathematical modeling.

• Gatekeeper mode (1.3 V): the noisy instability completely vanishes, replaced by a massive *R*_ct_ semi-circle. The polymer reaches its absolute maximum oxidation state, undergoing profound structural rigidification and generating an insurmountable electrostatic and steric barrier, effectively “sealing” the pores.

### Spectroelectrochemical validation of the switchable states

To optically validate the potential-dependent electronic transitions governing the different operational modes of the bio-hybrid device, UV-Vis-NIR spectroelectrochemistry was performed on the oligo-BT_2_T_4_@eggshell network (Fig. S5, SI). The bare biomineral scaffold exhibits no significant absorption, confirming its role as an optically transparent structural support.

At 0 V and 0.7 V (passive filter mode), the spectra are nearly identical, dominated by the π−π* transition in the visible region (∼400–500 nm). This indicates that at 0.7 V the bulk polymer matrix largely retains its neutral-like conjugated architecture, preserving the native chiral cavities required for pure enantioseparation without triggering chemical interconversion.

However, stepping the potential to 1.1 V (reactive mode) induces a dramatic optical modulation. The visible π−π* band undergoes significant bleaching, accompanied by the abrupt emergence of a broad, intense absorption band in the near-infrared (NIR) region spanning from 650 nm to beyond 1300 nm. This is the unequivocal optical signature of p-doping, corresponding to the generation of highly electrophilic polarons (radical cations) along the conjugated backbone. This observation perfectly corroborates our mechanistic rationale: the appearance of these charged polaronic states provides the necessary catalytic microenvironment to drive the surface-templated tautomerization and inversion of the carvone stereocenter.

Finally, at 1.3 V (Gatekeeper Mode), further bleaching and NIR evolution indicate the transition towards bipolaronic states. This extreme oxidation state maximizes the structural rigidity and electrostatic charge of the chiral tracks, providing the severe steric hindrance responsible for the near-total retention of the analytes observed during the continuous-flow filtration.

### Electrically switchable chiral separation in continuous flow

**Fig. 4:**
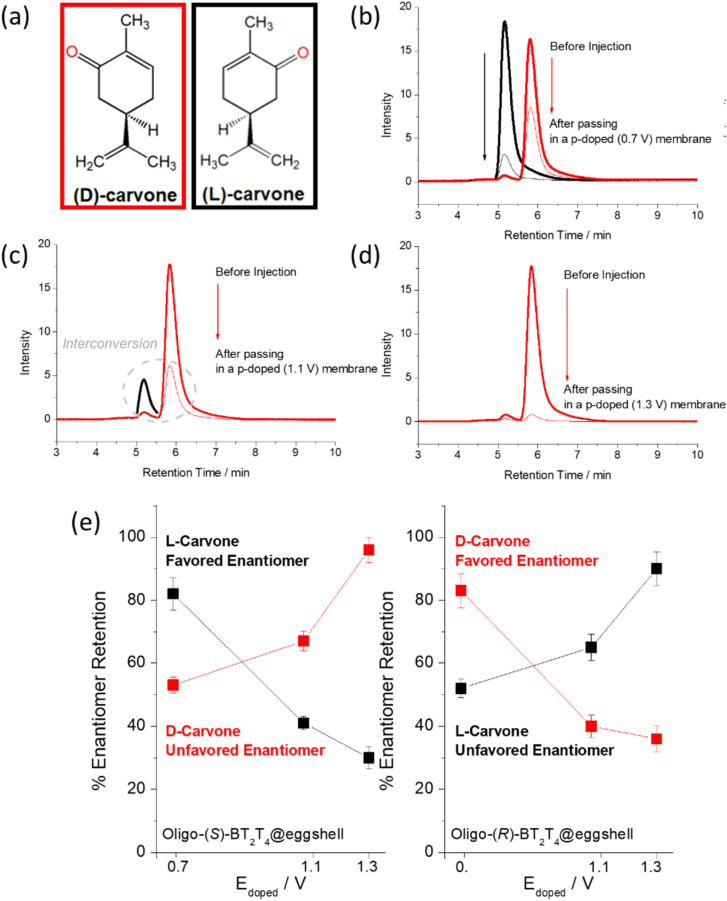
Electrically switchable chiral separation and solid-state electrocatalytic interconversion of carvone enantiomers (a) using the oligo-(*S*)-BT_2_T_4_@eggshell membrane. (b–d) HPLC chromatograms monitoring the permeation of enantiopure l-carvone (black traces) and d-carvone (red traces) before and after passing through the bio-hybrid membrane poised at applied DC biases of (b) 0.7 V (passive filter mode), (c) 1.1 V (reactive mode), and (d) 1.3 V (steric gatekeeper mode). (e) Comparative plot illustrating the perfectly mirrored retention efficiency for d- (in red) and l-carvone (in black) as a function of the applied potential (*E*_doped_/*V*) when utilizing the oligo-(*S*)-BT_2_T_4_@eggshell (left side) *versus* an oligo-(*R*)-BT_2_T_4_@eggshell (right side). Error bars in panel (e) represent the standard deviation for triplicate measurements (*n* = 3). This rigorous mirror-image symmetry unequivocally confirms the structural stereospecific origin of the dynamic gating effect.

To empirically evaluate this electrical switchability, the bio-hybrid membranes were mounted in a custom-built, miniaturized continuous-flow separation cell (Fig. 1c), minimizing solvent consumption and utilizing heptane as a green extraction medium. The HPLC analysis of the passing fractions (Fig. 4b–e) perfectly corroborated the mechanistic transitions implied by the EIS data. At a moderate anodic bias of 0.7 V (Fig. 4b) the membrane operates strictly through differential thermodynamic affinity.

The (*S*)-membrane accurately recognizes the complementary spatial geometry of l-carvone, trapping it within the matrix (87% retention efficiency), while the structurally mismatched d-carvone permeates unhindered (only 43% retention). Elevating the potential to 1.3 V (Fig. 4d) completely inverts the membrane's operational logic. The excessive *p*-doping induces the previously described high-impedance sealing effect. The unfavored d-carvone is now subjected to near-absolute forced retention (99%), while the retention of the initially favored l-carvone drops dramatically to 45%. The applied potential elegantly upgrades the membrane from a passive selective filter to a high-efficiency chiral barricade targeted specifically against the unfavored species. Most remarkably, when the membrane is poised at the intermediate potential of 1.1 V (Fig. 4c) it enters a hyper-reactive regime that represents a departure from simple physical filtration. As suggested by the kinetic instability observed in the EIS profiles (Fig. 3), this potential triggers the active electrocatalytic interconversion of the enantiomers. At this specific bias, the high-energy interaction between the oxidized chiral selector and the unfavored enantiomer (*e.g.*, d-carvone for the (*S*)-membrane) forces a structural correction, actively transforming the unfavored species into its favored antipode (l-carvone). This reactive resolution allows the system to bypass the 50% yield limitation inherent to passive thermodynamic filters, effectively upgrading the operational logic of the device from a selective sieve to a solid-state electrocatalytic reactor. By utilizing only localized electrical perturbation, the membrane begins to ‘correct’ the racemic equilibrium, setting the stage for the high-purity outcomes achieved in the subsequent multi-pass iterative process.

This mirrored symmetry in transport behavior is perfectly replicated when deploying the oligo-(*R*)-BT_2_T_4_@eggshell membrane (Fig. 4e).

At 0.7 V, d-carvone becomes the favored enantiomer (84% retention), while at 1.3 V, the (*R*)-membrane acts as an inverted gatekeeper, preferentially blocking the unfavored l-carvone (96% retention).

Crucially, this mirrored symmetry extends to the reactive regime at 1.1 V, where the oligo-(*R*)-BT_2_T_4_@eggshell membrane demonstrates an exactly opposite interconversion logic. While the (*S*)-counterpart converts d-carvone into l-carvone, the (*R*)-interface actively catalyzes the transformation of the now-unfavored l-enantiomer into the favored d-antipode. As illustrated in Fig. 4e, the inversion of the polymer's absolute configuration leads to a complete swap of the ‘favored’ and ‘unfavored’ roles.

At 1.1 V, the (*R*)-membrane exploits the high-energy state of the mismatched (*R*)-surface/(L)-analyte interaction to drive the electrocatalytic deracemization toward the d-form. This perfectly symmetrical response across all three potential-driven modes confirms that the device's operational logic, whether acting as a filter, a reactor, or a gatekeeper, is dictated by the precise stereochemical match between the analyte and the inherently chiral backbone.

To conclusively prove that this effect is governed solely by the inherently chiral backbone, rigorous control experiments were executed using an oligo-Racemic-BT_2_T_4_@eggshell membrane (successful polymer integration confirmed in Fig. 2c). The racemic membrane exhibits absolutely no preferential retention (∼51–52% for both enantiomers at 0.7 V, Fig. S6a). As the applied potential is increased to 1.1 V (Fig. S6b) and 1.3 V (Fig. S6c), a global increase in non-specific retention is observed, reaching values around 73–74%. This trend, shown in the linear progression of the retention plot, is attributed to the physical tightening and increased oxidation of the polymer mesh, which enhances the overall barrier properties of the membrane.

However, throughout this potential range, the retention difference between the two enantiomers remains almost zero. This ‘enantiomerically blind’ behavior definitively proves that the dynamic gating and the reactive interconversion seen in the enantiopure systems are not artifacts of non-specific electrostatic interactions, but are intrinsically dependent on the presence of ordered, continuous stereocenters within the inherently chiral backbone.

### Solid-state electrocatalytic deracemization in continuous flow

**Fig. 5:**
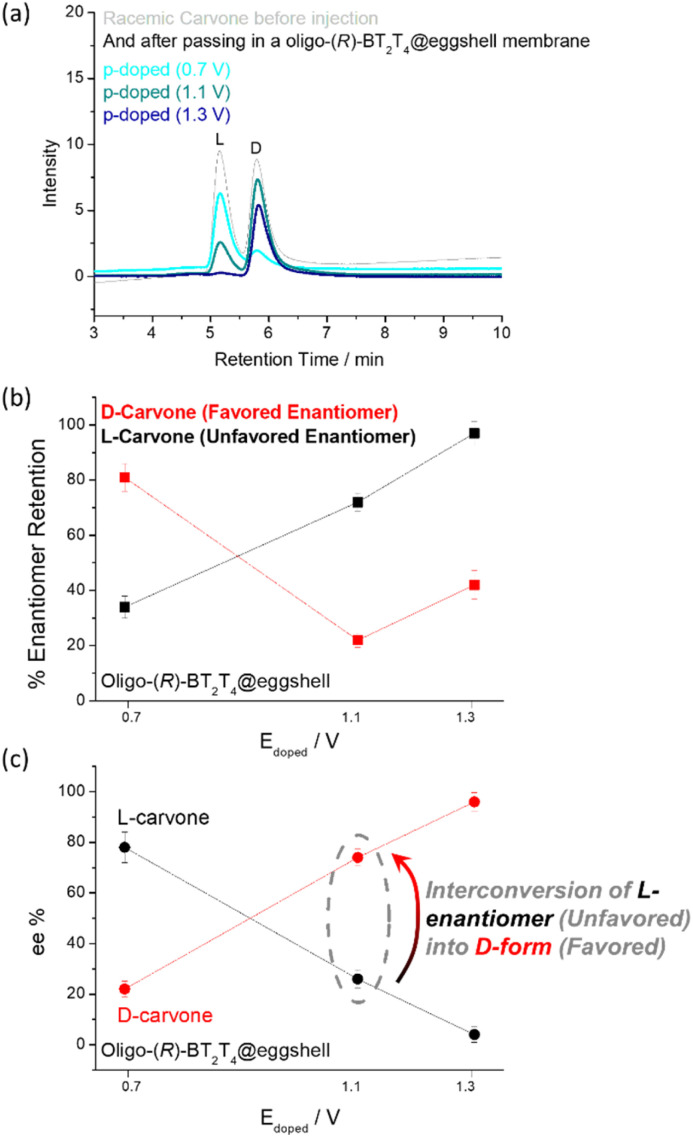
Solid-state electrocatalytic deracemization and potential-tuned gating of a racemic carvone using the oligo-(*R*)-BT_2_T_4_@eggshell membrane. (a) HPLC chromatograms tracking the continuous-flow processing of a 50 : 50 racemic carvone feed (grey trace) through the membrane at different applied DC biases: 0.7 V (light blue), 1.1 V (teal), and 1.3 V (dark blue). The profiles show the progressive depletion of the unfavored enantiomer and the modification of the permeate composition. (b) Comparative plot of retention efficiency for the favored d-carvone (red squares) and unfavored l-carvone (black squares). The device transitions from a passive thermodynamic filter at 0.7 V (retaining 81% of D and 34% of L) to an absolute steric gatekeeper at 1.3 V, where it effectively “seals” the pores against the unfavored l-enantiomer (97% retention). (c) Evolution of enantiomeric excess (ee%) in the collected permeate as a function of the applied potential (*E*_doped_). The dashed oval highlights the hyper-reactive 1.1 V regime, where the intense electrical perturbation actively catalyzes the electrocatalytic interconversion of the unfavored l-enantiomer directly into the favored D-form. This dynamic behavior confirms that the membrane's operational logic can be seamlessly switched between selective separation and active asymmetric synthesis solely by tuning the external electrical bias. Error bars in panel (b) and (c) represent the standard deviation for triplicate measurements (*n* = 3).

The most interesting result of this study emerged when the bio-hybrid membrane was precisely tuned to the intermediate, kinetically unstable potential of 1.1 V and challenged with a strict racemic mixture of carvone (50% L/50% D) (Fig. 5a–c).

At 0.7 V, the membrane merely filters the racemate based on primary affinity (retaining 81% of D and only 34% of L). At 1.3 V, it acts as a selective solid wall (retaining 97% of L and 42% of D). However, adjusting the bias to the reactive 1.1 V regime drastically alters the chemical landscape. Driven by the high energy state of the mismatched surface interaction, the membrane actively and forcibly “corrects” the racemate, electrocatalytically transforming the unfavored L-form directly into the favored D-form with an enantiomeric excess (% ee) of 74% for the d-carvone (Fig. 5c).

**Fig. 6:**
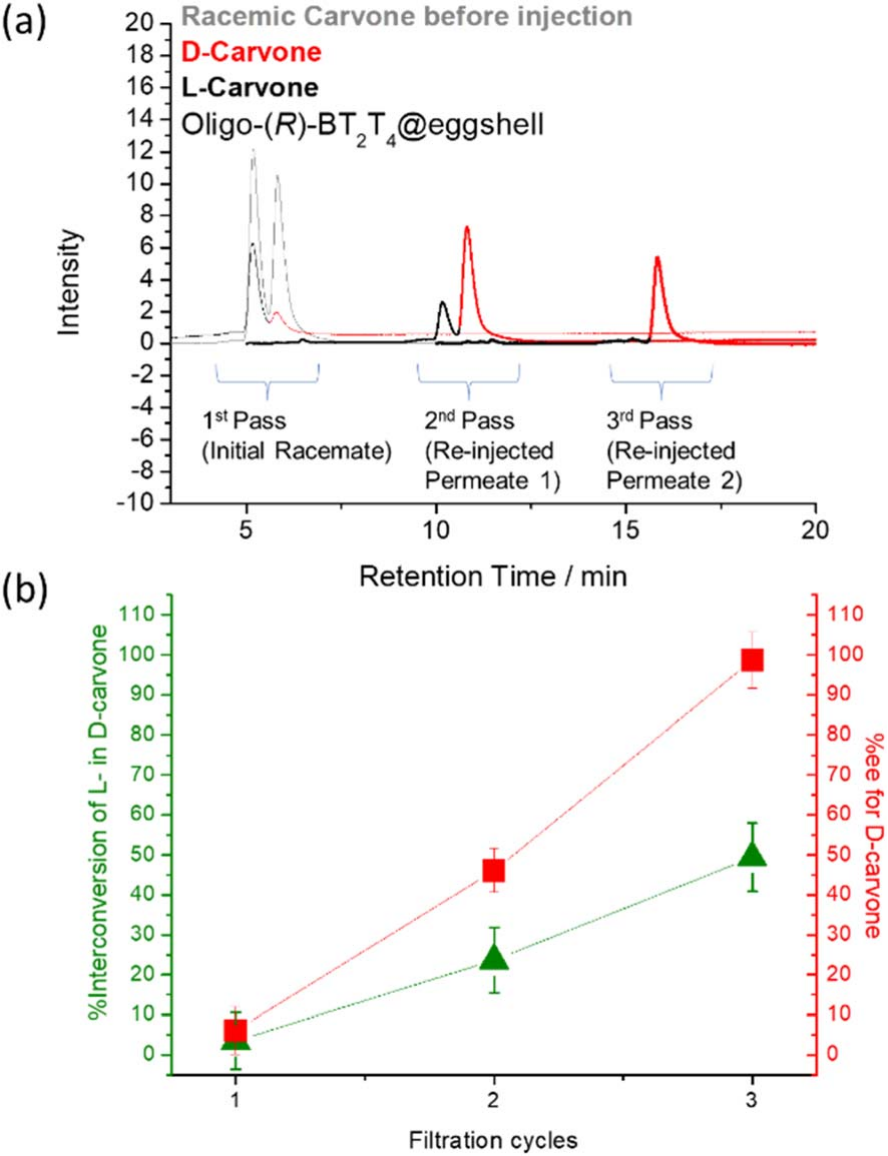
Quantitative multi-pass deracemization of carvone utilizing the oligo-(*R*)-BT_2_T_4_@eggshell bio-hybrid reactor at 1.1 V. (a) Iterative HPLC chromatograms monitoring the continuous-flow processing of an initial 50 : 50 racemic carvone feed (grey trace). The permeate from each pass was collected and re-injected into the membrane for three consecutive cycles. The progressive depletion of the unfavored l-carvone peak (black) and the concomitant amplification of the favored d-carvone peak (red) visually demonstrate the active electrocatalytic interconversion occurring at the hyper-reactive 1.1 V bias. (b) Quantitative evolution of the deracemization efficiency as a function of the filtration cycles. The enantiomeric excess (ee %) for the target d-carvone (red squares, right axis) shows a transformative progression from 6% in the first cycle to an exceptional 98.7% by the third cycle. This analytical improvement is strictly correlated with the solid-state electrocatalytic interconversion rate of l- into d-carvone (green triangles, left axis). By bypassing single-stage kinetic mass-transport limitations through a multi-pass configuration, the system successfully upgrades a low-value racemate into a highly pure single-enantiomer product (>99% ee) using only localized electrical potential. Error bars in panel (b) represent the standard deviation for triplicate measurements (*n* = 3).

While a single pass at 1.1 V achieves a partial reactive resolution (shifting the output composition to a ∼3.5% initial interconversion rate), thermodynamic constraints limit single-stage total conversion. To bypass the localized kinetic mass-transport limitations and engineer a system capable of absolute quantitative asymmetric synthesis, the bio-hybrid membrane was deployed in an iterative, multi-pass continuous-flow reactor configuration (Fig. 6). Each iterative cycle subjects the residual unfavored l-fraction to further intense electrochemical perturbation. The analytical results are profoundly transformative: the enantiomeric excess (ee%, Fig. 6b) of the favored d-carvone progressed from 6% in the first cycle, to 46% in the second cycle, and reached an exceptional 98.7% by the third cycle (peaking at 99.4% in optimized runs). As a result, the multi-pass continuous-flow process successfully drives the system toward a progressive deracemization, achieving a highly reproducible mean enantiomeric excess (ee%) of 98.7 ± 0.5% by the third filtration cycle, as illustrated in Fig. 6b.

This multi-pass regime conclusively demonstrates that the bio-hybrid interface successfully transforms a low-value racemic mixture into a highly pure, single-enantiomer product. By driving racemates to >99% ee utilizing solely localized electrical potential across a zero-cost, waste-derived biomineral scaffold, this approach offers an unprecedented, solvent-efficient alternative to traditional asymmetric syntheses and preparative chiral chromatography. To provide the ultimate chiroptical proof of this electrocatalytic transformation, the absolute configuration and structural integrity of the highly enriched d-carvone permeate were definitively corroborated by circular dichroism (CD) spectroscopy (Fig. S7). The exact match between the Cotton effect of our >99% ee permeate and the commercial enantiopure (d)-carvone standard provides incontrovertible evidence that the unfavored (l)-enantiomer was physically interconverted into the target (d)-enantiomer without undergoing any unwanted side reactions or electrochemical aromatization (*e.g.*, conversion to achiral carvacrol).

### Molecular mechanism of surface-electrocatalyzed interconversion

To elucidate the chemical pathway enabling the inversion of the C5 stereocenter under mild electrochemical conditions, and in light of the polaronic states just confirmed *via* spectroelectrochemistry, the molecular–level interactions at the bio-hybrid interface must be considered. The configuration switch from l- to d-carvone at 1.1 V necessarily requires a transient planarization of the stereogenic center, a process that is typically unfavorable in bulk solution without harsh acid/base catalysts. Here, the partially oxidized (p-doped) polaronic state of the oligo-BT_2_T_4_ matrix at 1.1 V acts as an effective heterogeneous electrocatalyst. The proposed mechanism proceeds *via* a surface-templated keto–enol/dienol tautomerization or a reversible single-electron transfer (SET) pathway. Upon adsorption onto the electrophilic, positively charged thiophene network, the carvone molecule experiences strong local polarization. This interaction dramatically weakens the localized C–H bonds, promoting a surface-mediated proton abstraction that generates a highly conjugated enolate/dienol or an allylic radical intermediate. During this transition, the C5 chiral center temporarily rehybridizes from a sp^3^ geometry to a planarized sp^2^ state. The subsequent asymmetric induction is strictly governed by the inherently chiral environment of the polymer matrix. The planarized intermediate is structurally confined within the asymmetric topology of the oligo-(*S*)-BT_2_T_4_ tracks, forming a transient diastereomeric surface complex where one enantiotopic face is heavily shielded. Consequently, stereospecific re-protonation is directed exclusively from the unhindered face, preferentially yielding the d-enantiomer. Irreversible rearrangement and aromatization into achiral carvacrol are successfully suppressed because the mild applied bias (1.1 V) and the protective biomineral microenvironment do not provide the overpotential required for extended skeletal dehydro-isomerization. This electrocatalytic, surface-confined pathway successfully bridges the gap between high enantioselective control and mild operational regimes.

### Operational stability and reusability

**Fig. 7:**
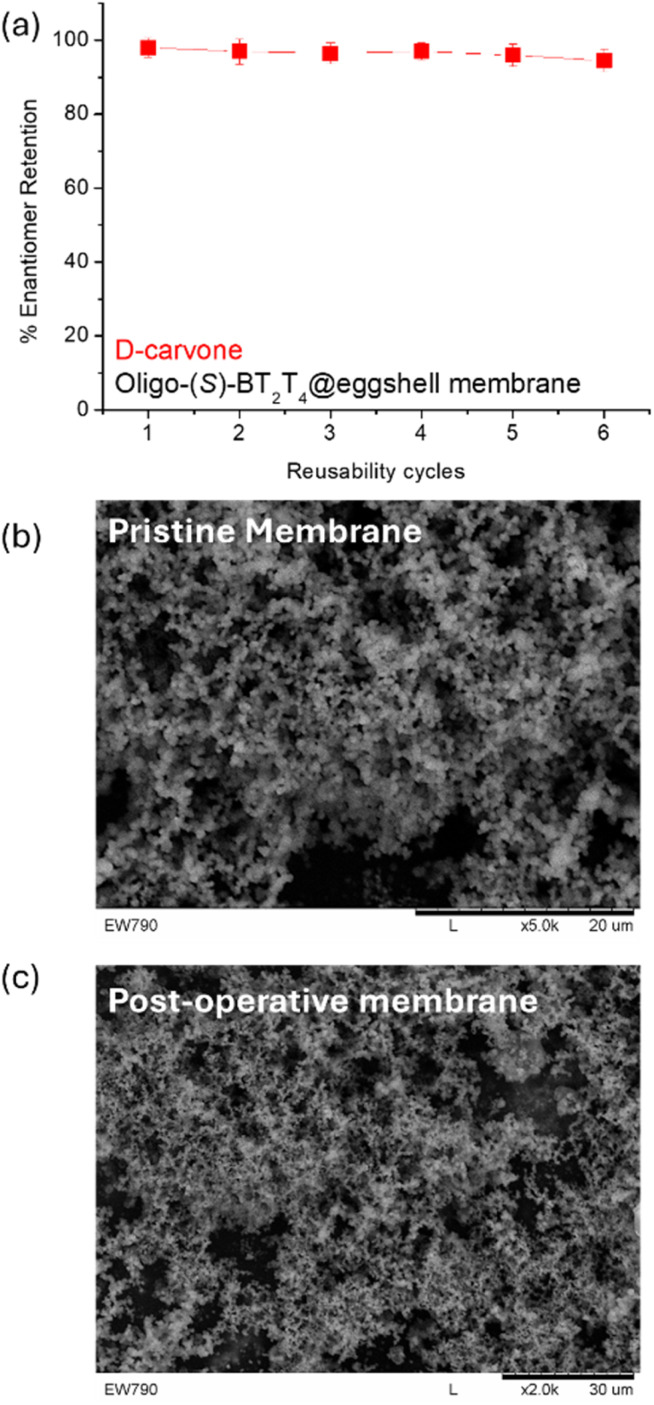
(a) Retention efficiency of d-carvone over six sequential filtration cycles using the same oligo-(*S*)-BT_2_T_4_@eggshell membrane p-doped at a constant 1.3 V. Error bars represent the standard deviation for triplicate measurements. SEM micrographs comparing the pristine membrane (b) and the post-operative membrane (c) after the stability test.

The long-term operational robustness and reusability of the bio-hybrid device were evaluated by subjecting the oligo-(*S*)-BT_2_T_4_@eggshell (p-doped at 1.3 V) membrane to six consecutive filtration cycles using d-carvone (Gatekeeper Mode). Crucially, between each sequential run, the membrane was regenerated through a standardized washing protocol using green extraction media (heptane) to ensure the complete removal of any previously retained analyte from the tortuous biomineral matrix (as detailed in the experimental section). As illustrated in Fig. 7, the enantiomer retention efficiency remains exceptionally stable, consistently between 95–98% across all cycles with minimal statistical deviation. This sustained performance is further corroborated by post-operative SEM analysis, which reveals that the hierarchical micro-porosity and the tortuous labyrinthine network of the biomineral scaffold remain perfectly intact even after prolonged exposure to continuous flow. The absence of polymer delamination or biomineral erosion confirms that the conformal, hydrophobic oligo-BT_2_T_4_ coating acts as an effective protective shield. This “core–shell” architecture successfully stabilizes the acid-sensitive calcium carbonate core against the extraction media and the applied electrical stress, proving that the binder-free assembly provides sufficient mechanical and electrochemical durability for preparative chiral applications.

### Green metrics and sustainability assessment

To quantitatively contextualize the environmental advantages of the proposed bio-hybrid device, the system was evaluated against standard Green Chemistry and Green Analytical Chemistry (GAC) metrics, specifically focusing on Atom Economy (AE), energy consumption, processing time, and a quantitative Environmental Factor (*E*-factor). In conventional chiral resolution, such as preparative HPLC or classic diastereomeric salt crystallization, the maximum theoretical yield of the desired enantiomer from a racemic mixture is strictly limited to 50%. Consequently, the intrinsic Atom Economy of a simple physical separation is capped at 50%, as the remaining unfavored antipode is inherently discarded as chemical waste.

The electrocatalytic deracemization regime operating at 1.1 V effectively overcomes this limitation. By actively interconverting the unfavored l-carvone into the valuable d-enantiomer, our continuous-flow reactor pushes the theoretical Atom Economy of the process to 100%. However, while this 100% efficiency represents the thermodynamic limit, a single-pass operation yields an initial interconversion of approximately 3.5%.

Therefore, to achieve an optical purity exceeding 99% ee, a multi-pass recycling mode is required, which demands a total processing time of approximately 12 minutes for a 40 µL batch. Notably, this interconversion is driven exclusively by electrical potential. By utilizing electrons as zero-mass reagents, the system circumvents the use of stoichiometric chemical oxidants, reductants, or toxic metal catalysts typically required in standard asymmetric syntheses, significantly reducing the overall process mass intensity (PMI). Due to the ultra-low current required to drive the solid-state electrocatalysis on the highly conductive oligo-BT_2_T_4_ network (average operating current ∼50 µA at 1.1 V), the specific electrical energy consumption (*E*_cons_) was determined to be exceptionally low (1.2 × 10^−4^ kW h per gram of refined product).

From a solvent consumption perspective, classical preparative chiral chromatography relies on the continuous elution of large volumes of organic mobile phases (often toxic mixtures containing hexane, isopropanol, or halogenated solvents), generating a high *E*-factor (kg of waste per kg of product). In contrast, the miniaturized analytical setup presented herein operates in a solvent-efficient micro-flow regime. Taking into account the 1.5 mL of heptane, deliberately chosen as a green alternative to standard aliphatic extraction solvents, used for system flushing and product recovery, the quantitative *E*-factor was calculated to be approximately 75.

Finally, the sustainability of the stationary phase material itself represents a significant advancement. Commercial chiral stationary phases (CSPs) require energy-intensive manufacturing, relying on highly engineered silica beads coated with derivatized petroleum-based polymers or complex polysaccharides. Our strategy avoids this environmental burden by directly upcycling an abundant, zero-cost food industry bio-waste (calcium carbonate from eggshells) utilizing a binder-free approach. To provide a balanced and rigorous assessment, these quantitative metrics are summarized and directly compared with conventional preparative chiral HPLC in Table S2.

## Conclusions

The successful, binder-free integration of inherently chiral BT_2_T_4_ oligomers onto globally abundant, upcycled eggshell biomineral waste (CaCO_3_) marks a fundamental paradigm shift in the design and execution of enantioselective analytical devices. The results presented herein systematically dismantle the traditional reliance on passive, static, and resource-intensive chromatographic media. By utilizing a zero-cost, environmentally sustainable inorganic scaffold, which intrinsically provides a highly complex, tortuous geometry acting as a “structural multiplier”, the spatial resolution and absolute retention capabilities of the chiral selector are magnified by orders of magnitude. Furthermore, the conformal electrodeposition of the highly hydrophobic π-conjugated polymer generates a protective core–shell architecture. This physical encapsulation effectively shields the acid-sensitive biomineral core, expanding the operational pH window of the waste-derived scaffold and enabling robust chiral analysis even in hostile acidic environments.

More significantly, the empirical demonstration of a potential-tunable chiral interface permanently blurs the historically rigidly defined boundaries between physical separation sciences and synthetic electrocatalysis. The ability to dynamically manipulate a single, low-cost bio-hybrid membrane *via* direct current to act selectively as a passive thermodynamic affinity filter (0.7 V), an active deracemizing solid-state reactor (1.1 V), or an absolute steric gatekeeper (1.3 V) provides unprecedented operational flexibility for continuous-flow monitoring, diagnostic sensing, and targeted purification.

Furthermore, the pivotal discovery that kinetic instability, routinely observed and discarded as instrumental noise in standard EIS measurements, can serve as a direct, non-destructive, *in situ* probe for monitoring active enantiomeric interconversion offers a highly novel diagnostic tool for tracking dynamic stereochemical processes at solid–liquid interfaces.

The successful translation of this reactive regime into a miniaturized, solvent-efficient multi-pass continuous-flow loop demonstrates a highly scalable blueprint for quantitative asymmetric synthesis. Operating with microliter volumes and utilizing green extraction media (heptane), the system actively drove strict racemic mixtures to an exceptional >99% enantiomeric excess using solely minimal localized electrical voltage. This unique architectural approach provides a highly efficient, reagent-free, and genuinely sustainable alternative to traditional, chemically wasteful deracemization techniques. Ultimately, these findings establish the definitive foundation for a new class of smart, electrically switchable molecular sieves with vast applications ranging from decentralized, point-of-care pharmaceutical purification to advanced green synthetic organic chemistry.

## Author contributions

Malinee Niamlaem: methodology, writing – original draft, visualization, validation, data curation, formal analysis, investigation, conceptualization. Sara Grecchi: writing – review & editing, writing – original draft, methodology, formal analysis, data curation, conceptualization. Mariangela Longhi: writing – review & editing, BET formal analysis, data curation. Serena Arnaboldi: writing – review & editing, funding acquisition, project administration, resources, supervision, conceptualization.

## Conflicts of interest

The authors declare that there is no conflict of interest in this study.

## Supplementary Material

RA-OLF-D6RA03698H-s001

## Data Availability

The data that support the findings of this study are available from the corresponding author upon reasonable request. Supplementary information (SI): detailed characterization and experimental datasets, including multi-scale SEM micrographs, comparative FTIR spectra, cyclic voltammograms, control experiment HPLC profiles, and circular dichroism (CD) spectra. See DOI: https://doi.org/10.1039/d6ra03698h.
