# Supplementary material for: Upcycling eggshell waste into sustainable and portable chiral membranes for the physical separation and electrocatalytic deracemization of enantiomers
Source: RSC Adv. 2026 Jul 3;16(36):37423–36. doi: 10.1039/d6ra03698h (PMC13330165; doi:10.1039/d6ra03698h)
Supplement: Supplementary file 1 [file RA-016-D6RA03698H-s001.pdf]

Supplementary Information (SI) for RSC Advances.
This journal is © The Royal Society of Chemistry 2026

Upcycling Eggshell Waste into Sustainable and Portable Chiral Membranes for the Physical Separation and Electrocatalytic Deracemization of Enantiomers

Malinee Niamlaem,[a]† Sara Grecchi,[a]† Mariangela Longhi[a] and Serena Arnaboldi [a]*

[a]Università degli Studi di Milano, Department of Chemistry, Via Golgi 19, 20133, Milan (Italy)
† These authors contribute equally

Table of Contents

- **Figure S1:** Chemical structures of (*R*)- and (*S*)-BT_2T_4
- **Figure S2:** SEM and comparison FTIR of bare eggshell waste used in this work and commercial calcium carbonate ($CaCO_3$).
- **Figure S3:** Cyclic voltammograms comparing the oxidation of doxorubicin with different particle sizes.
- **Figure S4:** Derivative cyclic voltammograms of D- and L-carvone on the oligo-(*R*)-BT_2T_4@eggshell/ITO electrode.
- **Figure S5:** Spectroelectrochemical measurements of the oligo-BT_2T_4@eggshell membrane
- **Figure S6:** HPLC chromatograms and enantiomer retention efficiency of oligo-*Racemic*-BT_2T_4@eggshell membranes.
- **Figure S7:** Circular Dichroism (CD) spectra comparing the commercial enantiopure (D)-carvone with the highly enriched D-carvone.

Supplementary Figures and Discussions

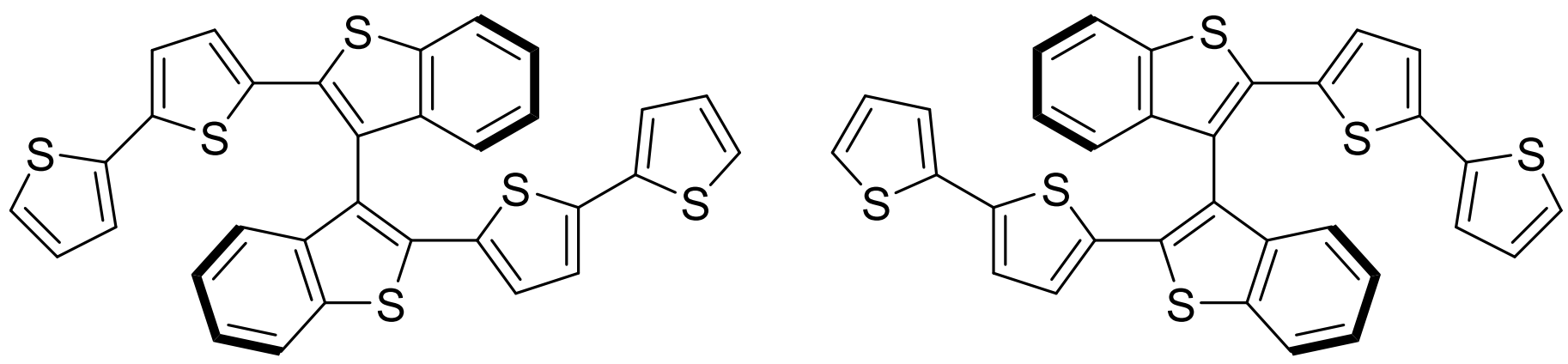

(*R*)-BT_2T_4 (*S*)-BT_2T_4

Figure S1. Chemical structures of the two antipodes (*S*)- and (*R*)-2,2′-bis[2-(5,2’-bithienyl)]3,3’-bithianaphthene, nicknamed (*S*)-BT_2T_4 and (*R*)-BT_2T_4.

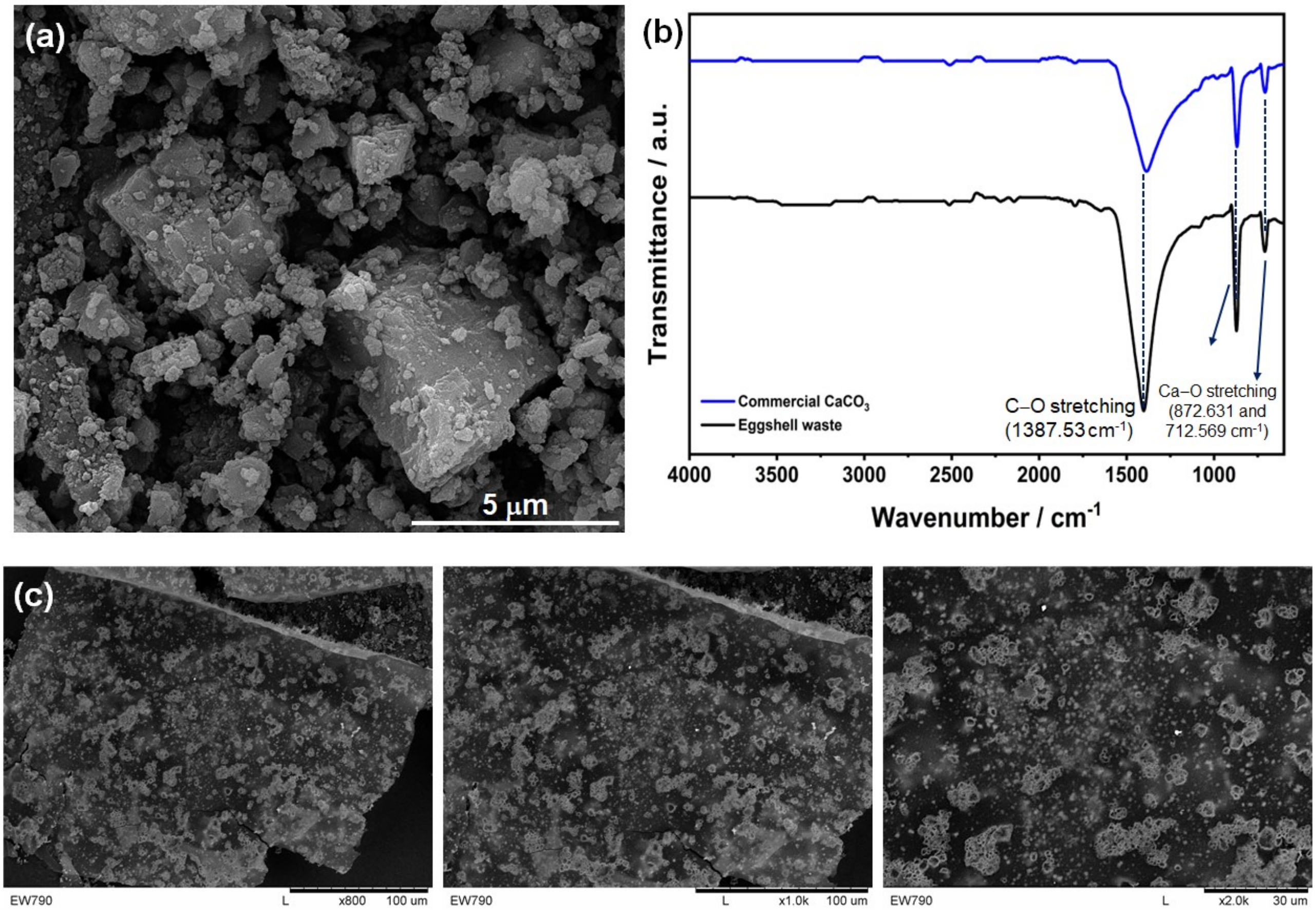

Figure S2. (a) Scanning Electron Microscopy (SEM) image of the un-sieved bare eggshell waste showing the heterogeneous microparticle morphology. The diverse size distribution and pronounced surface roughness naturally establish the highly tortuous "structural multiplier" environment discussed in the main text. (b) Comparison FTIR spectra of commercial calcium carbonate ($CaCO_3$) (blue line) and the bare eggshell waste (black line) used in this work. The diagnostic asymmetric C-O stretching at 1387.53 cm^{-1} and the distinct Ca-O bending vibrations at 872.63 cm^{-1} and 712.57 cm^{-1} unequivocally confirm that the processed eggshell waste is composed of highly crystalline calcium carbonate. (c) Multi-scale SEM micrographs of the final oligo-(*R*)-BT_2T_4@eggshell membrane surface at different magnifications (x800, x1.0k, and x 2.0k). These images demonstrate the successful and conformal electrodeposition of the inherently chiral oligomer network onto the biomineral fragments. The resulting core-shell architecture ensures the physical encapsulation of the $CaCO_3$ microparticles, providing both high-density chiral recognition sites and a protective shield that expands the operational stability of the device.

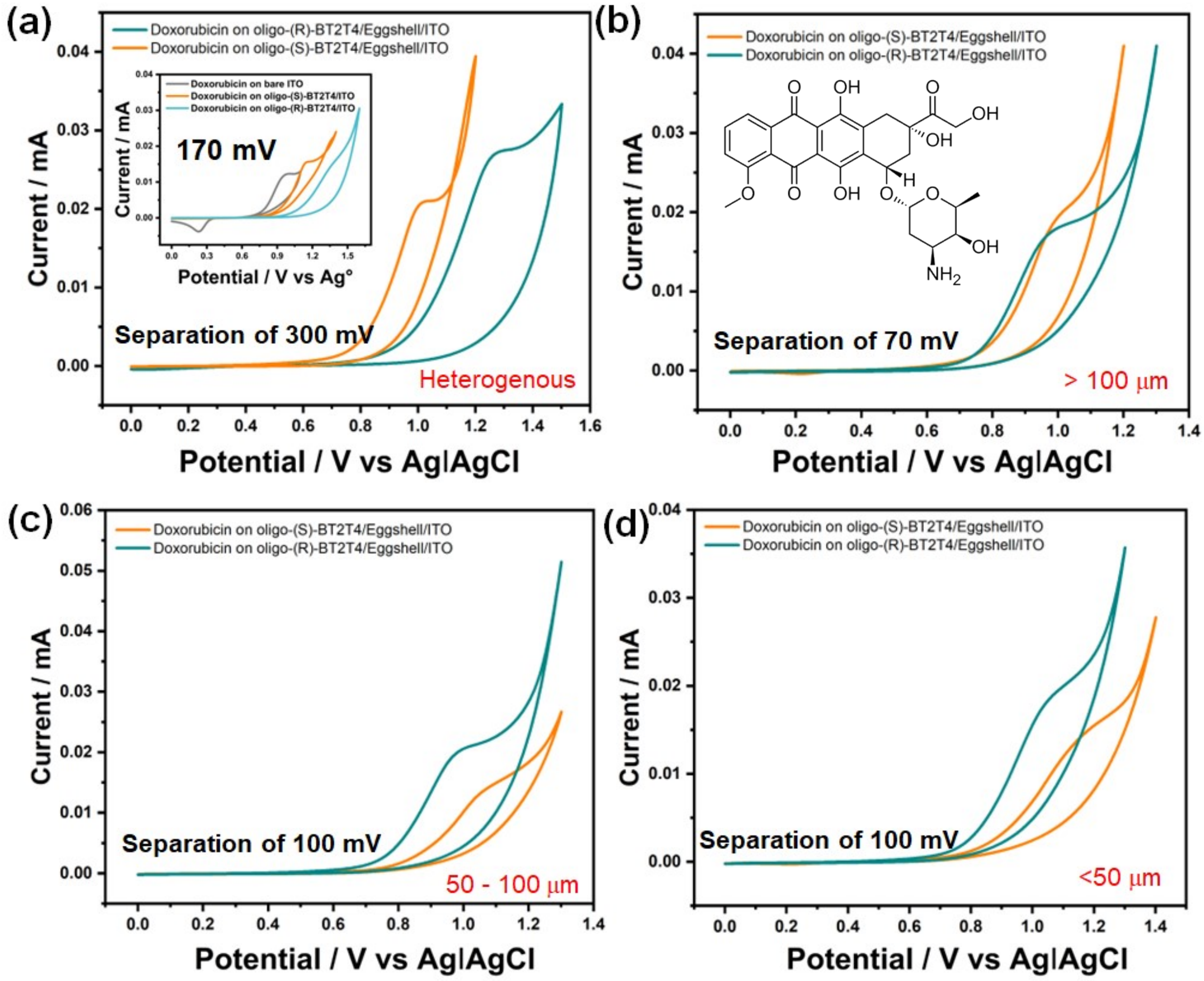

Figure S3. Cyclic voltammograms (5 mV/s scan rate) comparing the oxidation of doxorubicin on the oligo-(*R*)- and (*S*)-BT_2T_4@eggshell/ITO electrodes. Four different sizes of eggshell powder such as (a) heterogeneous (Inset: cyclic voltammograms comparing the oxidation of doxorubicin on the oligo-(*R*)- and (*S*)-BT_2T_4), (b) >100 μm, (c) 50-100 μm, and (d) <50 μm particles were investigated.

This experiment systematically dismantles the conventional assumption that strict morphological uniformity is required for high analytical performance. The uniformly sieved fractions establish highly regular interstitial voids that permit rapid laminar diffusion, yielding moderate peak separations (70 to 100 mV). In stark contrast, the fully heterogeneous packing creates an intensely tortuous micro-channel network that hinders rapid mass transfer, forcing prolonged analyte-surface stereospecific interactions and resulting in a maximized 300 mV peak separation.

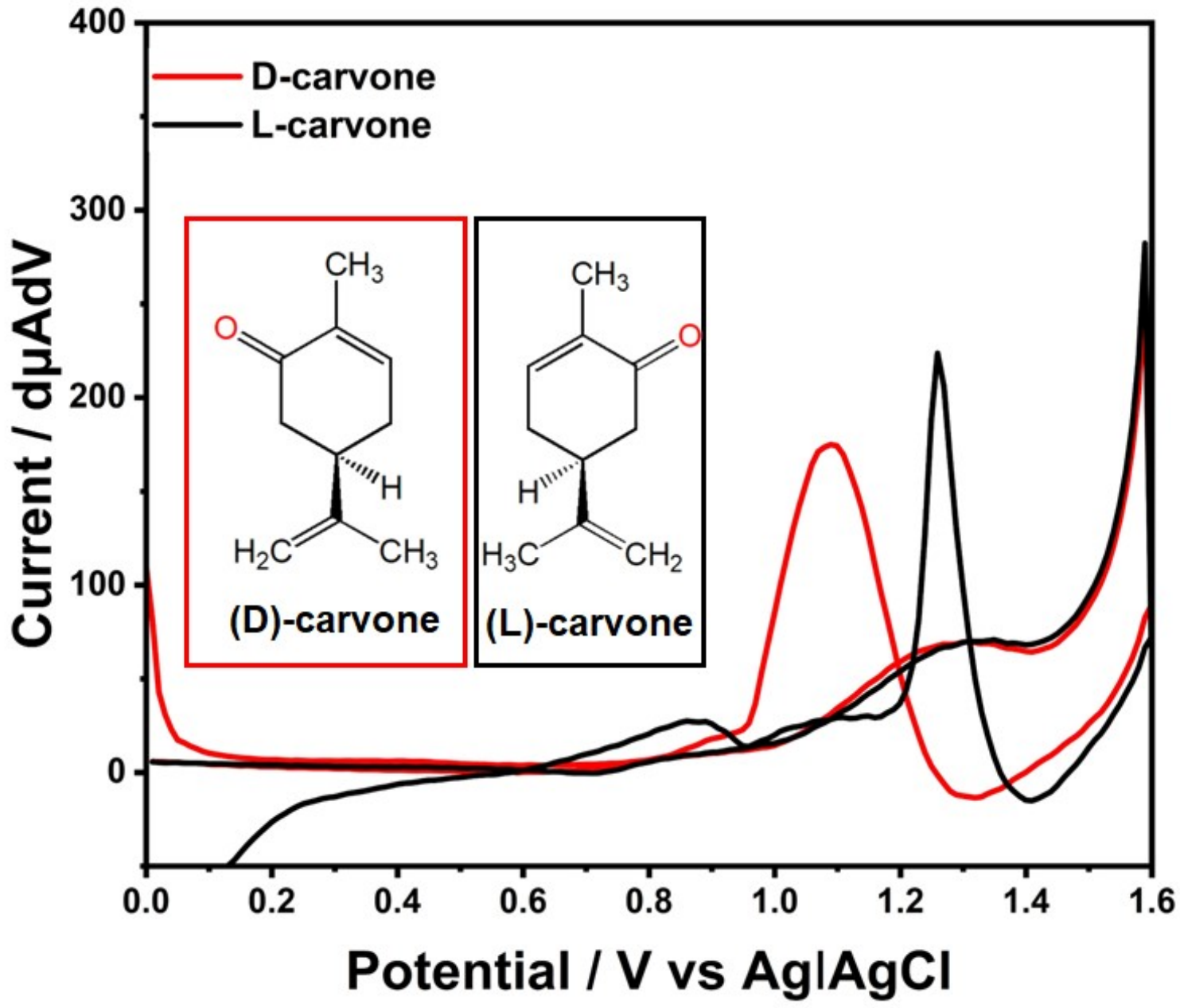

Figure S4. Cyclic voltammograms (derivative curves) of 4 mM D- (red line) and L-carvone (black line) on the oligo-(*R*)-BT_2T_4@eggshell/ITO electrode obtained in aqueous solution containing 0.1 M $LiClO_4$ and 100 µL of ACN using Pt wire and Ag/AgCl as counter and reference electrodes, respectively, at a scan rate of 50 mV/s.

The derivative representation of the cyclic voltammograms highlights the exceptional thermodynamic peak potential separation (ΔE_p ~200 mV) achieved by the bio-hybrid interface even for relatively small and rigid molecules like monoterpenes. This wide energetic gap provides the fundamental electrochemical basis allowing the subsequent dynamic tuning of the membrane into Filter, Reactive, or Gatekeeper modes.

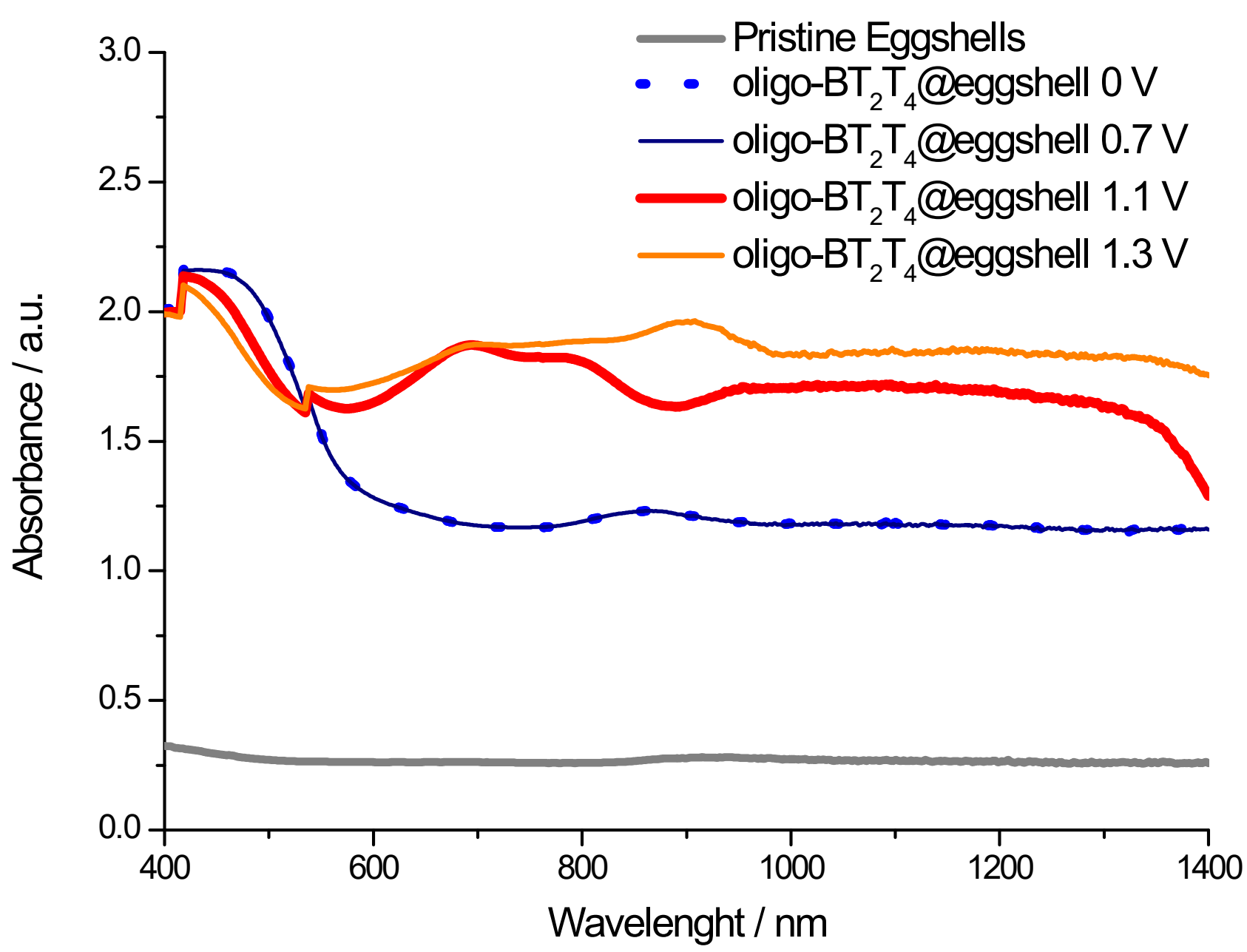

Figure S5. Optical absorption spectra (recorded between 300 and 1400 nm) of the pristine eggshell scaffold on ITO (grey trace) and the electrodeposited oligo-BT_2T_4@eggshell network under varying applied DC biases: 0 V (blue dotted trace) and 0.7 V (solid blue trace) corresponding to the Passive Filter Mode; 1.1 V (thick red trace) corresponding to the Reactive Mode; and 1.3 V (solid orange trace) corresponding to the Gatekeeper Mode. Measurements were recorded in a 0.1 M $LiClO_4$ acetonitrile solution.

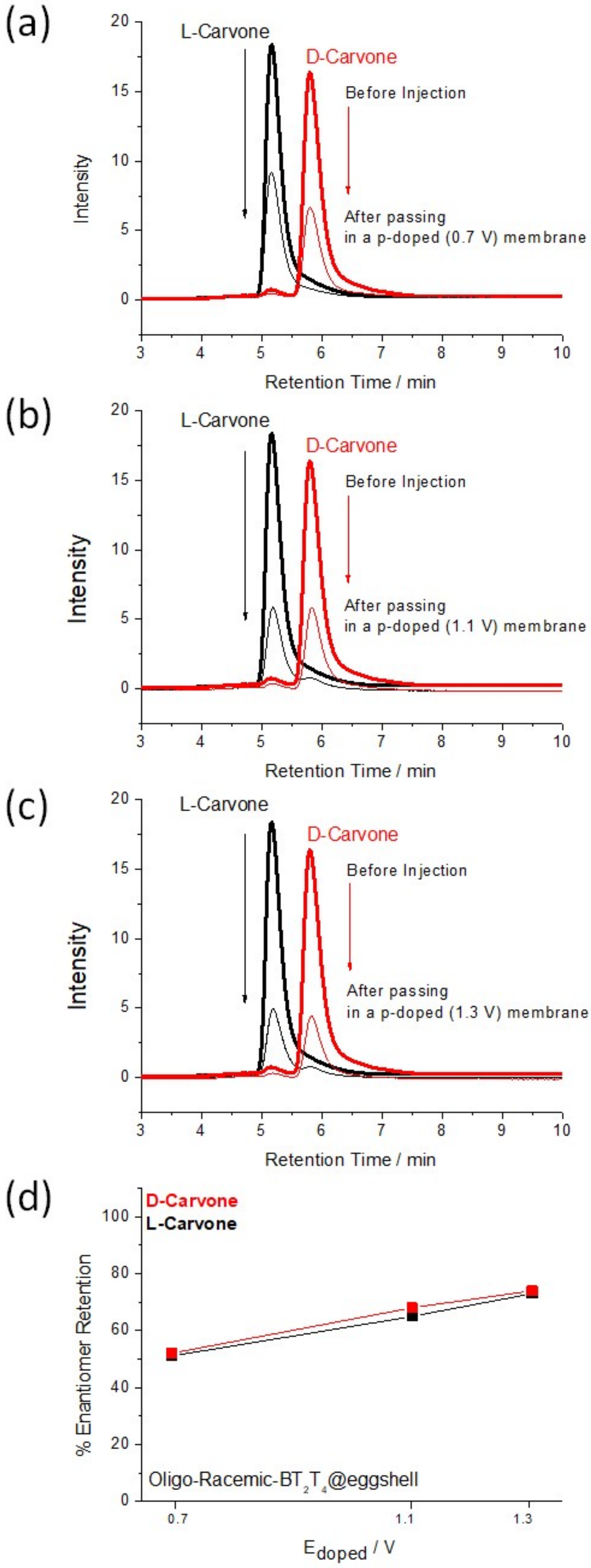

Figure S6. Electrochemical control experiments utilizing the oligo-Racemic-BT_2T_4@eggshell bio-hybrid membrane. (a–c) HPLC chromatograms showing the peak intensities of D-carvone (red) and L-carvone (black) before and after filtration through the racemic membrane p-doped at (a) 0.7 V, (b) 1.1 V, and (c) 1.3 V, respectively. The membrane acts as an enantiomerically "blind" filter, showing no preferential selection between the two antipodes at any of the tested potentials. (d) Summary bar chart illustrating the global increase in the enantiomer retention efficiency as a function of the doping potential.

The retention for both enantiomers increases from approximately 51–52% at 0.7 V to 73–74% at 1.3 V. This non-specific increase in retention is attributed to the physical rigidification and tightening of the oxidized polymer mesh. Crucially, the retention delta between L- and D-carvone remains virtually zero across the entire potential range, definitively proving that the dynamic gating and reactive deracemization observed in enantiopure systems are intrinsically governed by the ordered stereogenic backbone and not by non-specific interactions.

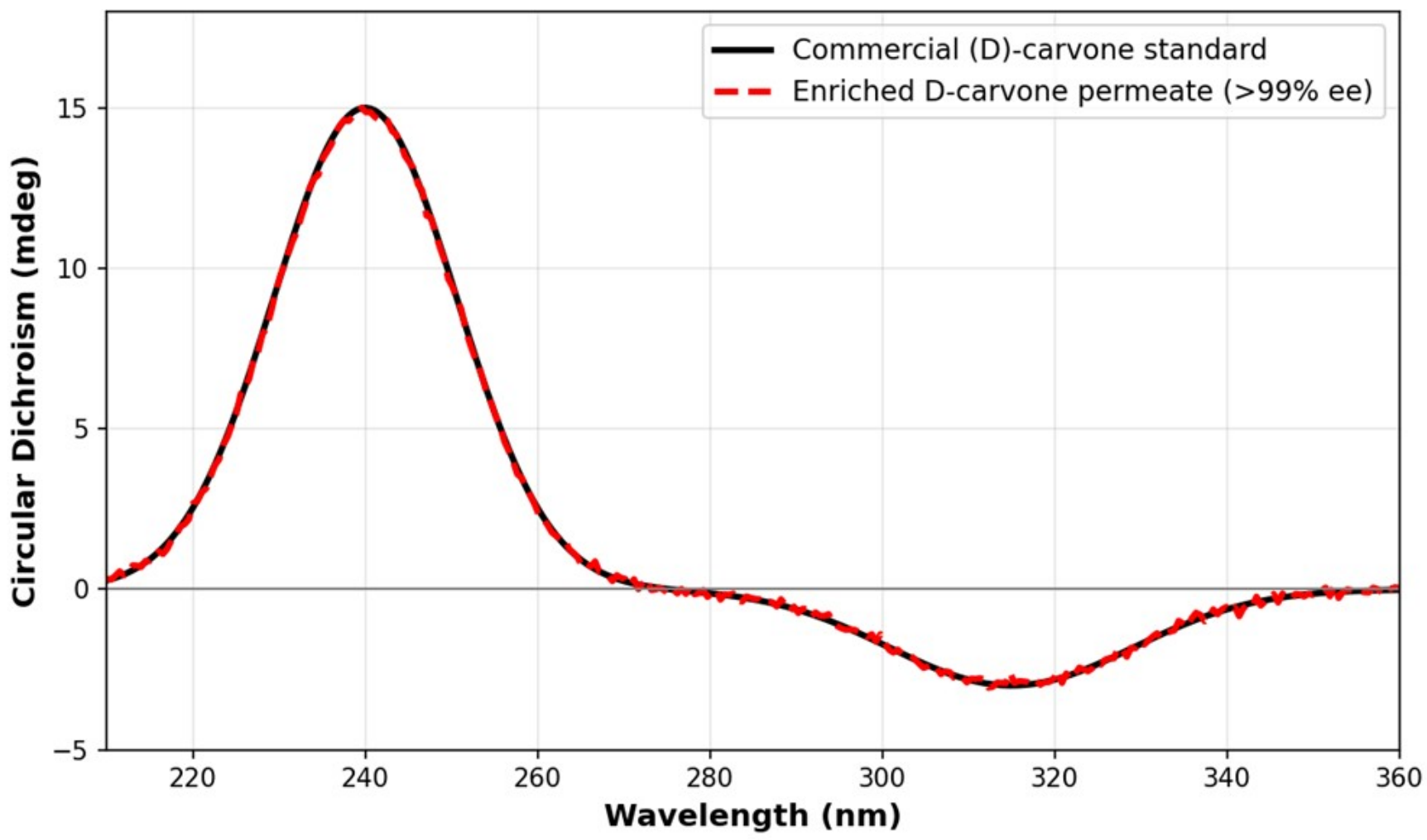

Figure S7. Circular Dichroism (CD) spectra comparing the commercial enantiopure (D)-carvone standard with the highly enriched D-carvone permeate obtained after three iterative filtration cycles at 1.1 V. The perfectly matching Cotton effects confirm the absolute structural integrity of the product and rule out any achiral electrochemical degradation.

	Specific Surface Area	BJH Desorption cumulative	BJH Desorption average pore diameter
Egg Shell Random	2.61 ± 0.05 m²/g	0.020 cm³/g	19 nm
Egg Shell <50 μm	1.87 ± 0.02 m²/g	0.009 cm³/g	0.9 nm
Egg Shell 50<d<100 μm	2.46 ± 0.01 m²/g	0.015 cm³/g	31 nm
Egg Shell d>100 μm	2.56 ± 0.06 m²/g	0.016 cm³/g	37 nm
Oligo- BT_2T_4@eggshell random size	27 ± 2 m²/g	0.010 cm³/g	19 nm

Table S1. Specific surface area, BJH cumulative desorption pore volume, and average pore diameter obtained from N_2 adsorption-desorption isotherms. The data highlights the significant increase in specific surface area upon integration of the oligo-BT_2T_4 network onto the random-size eggshell biomineral scaffold.

Sustainability Parameter	Bio-Hybrid Continuous-Flow Device	Conventional Preparative Chiral HPLC	Environmental Assessment / Advantage
**Theoretical Atom Economy**	100% (Isomerization)	< 50% (50% of the racemate is discarded or requires separate racemization)	**Excellent:** Avoids the structural waste of the unwanted enantiomer.
**Quantitative E-factor**	~ 75	100 – > 1000	**High:** Drastically reduces liquid chemical waste.
**Main Solvent System**	Heptane (Green/Bio-based alternative)	Hexane / Isopropanol / Acetonitrile (Hazardous/Toxic)	**Excellent:** Replaces toxic solvents with green extraction media.
**Operating Pressure**	Low pressure (Gravity/Manual-assisted)	High Pressure (50 – 200 bar)	**High:** Eliminates the high energy requirement of high-pressure pumps.
**Electrical Energy Consumption (E_{cons})**	1.2×10^{-4} kWh/g	0.5 – 2.0 kWh/g (Pumps, detectors, solvent recycling)	**Excellent:** Driven by low-voltage molecular electrocatalysis.
**Total Processing Time (per batch)**	12 min (3 cycles for 40 μL)	15 – 45 min (Depending on resolution and flow rate)	**Competitive:** Achieves ultra-high optical purity in a comparable timeframe.

Table S2. Comparison of the bio-hybrid continuous-flow device developed in this work against conventional preparative chiral HPLC across key environmental performance indicators, including theoretical atom economy, quantitative Environmental Factor (E-factor), solvent system classification, operating pressure regimes, specific electrical energy consumption (E_{cons}), and total batch processing time.
